# A Compendium of Medical Practice, Illustrated by Interesting and Instructive Cases, and by Practical Pathological, and Physiological Observations

**Published:** 1816-08

**Authors:** 


### PART II.

### COMPREHENSIVE ANALYTICAL REVIEW
OF
### MEDICAL LITERATURE.

"Tros, tyriusve, *nobis* nullo discrimine agetur."

*A Compendium of Medical Practice, illustrated by interesting and instructive Cases, and by Practical Pathological, and Physiological Observations.* By James Bedingfield, Surgeon; late Apothecary to the Bristol Infirmary. London, 1816. 8vo. pp. 309.

That Mr. Bedingfield attaches no trifling importance to his work is apparent in every page of it, and even on the cover, where *fifteen shillings* for 309 octavo pages, *without plates,* remind us of the sibylline books and the value set upon them! There is no danger, however, that the work before us shall share the fate of *any part* of the sibylline leaves; "Stationers Hall," in which the book is carefully entered, will preserve its contents from dispersion or piracy; while that laudable *œconomy,* which our government is *voluntarily* inculcating, will very effectually prevent the Medical Tarquins of the day from complying with the extravagant demands of the Bristol Sibyl.

After perusing the closely printed volume of Dr. Parry, every line of which is pregnant with information, and *there* seeing a price of fourteen shillings for 464 pages, we were not a little amused with the vanity of Mr. Beddingfield in affixing a sum to his lean volume which must deter even the lovers of medical literature for satisfying so unjust, so mercenary, and so ungenerous a demand on their pockets.

It is not our custom to "count the costs" upon many occasions; but we are induced to animadvert thus upon the *price* of the book, because we find it only a part of that system of arrogance which pervades the whole. Of this, the author himself was well aware.

"The author is aware, that in some parts of his volume, he has been betrayed into the adoption of an egotistical, in other parts of a *dictatorial* style." Preface ix.

But it is time to proceed to the work. Had it been entitled "Practical Remarks on Clinical Cases at the Bristol Infirmary," and written in a modest style, it would have escaped without censure; but its author has now thrown the gauntlet to criticism itself, and we hesitate not to take it up—our maxim being—

"Parcere subjectis et debellare *superbos*."

The alpha and omega of Mr. Bedingfield's professional reading, appear to be—Cullen's Nosology, and the London Medical Repository. Every *Chapter* in the work, of which, gentle reader, there are no less than *eighty-one!* (or, not quite four pages to a chapter) begins with a quotation from Cullen, while the respected contemporary Journal abovementioned, has the honour of furnishing a motto for the whole!

We shall proceed seriatim. Chap. 1. (p. 1 to 3.) "*On painful affections of the scalp.*" The first piece of *novelty* here, is advanced in bad English, "derangement in the functions of the *digestive organs* often precedes the local affection, and *they* (whether this pronoun applies to *derangement* or *affection,* it is equally ungrammatical) are uniformly aggravated by it." The *novelty* of this passage will be confirmed by the following:

"The *blue pill* unquestionably will, if properly administered, establish a more healthy action in the liver, and alimentary canal than any other remedy." "Fowler's solution ought to accompany its exhibition." End of chapter the first.

Chap. 2. *Tinea capitis.* (page 4 to 6) "The same remedies which prove so efficacious for the cure of psora, are *equally* so in this malady." Sulphur and tar are the favourite remedies in the Bristol Infirmary, and are said by Mr. B. to "seldom fail."

Chap. 3. *Hydrocephalus externus.* (page 7.) "This disease uniformly and inevitably proves, sooner or later, fatal." Mr. B. offers no hope nor plan of treatment.

Chap. 4. *Hydrocephalus internus.* (page 8 to 28.) Long as is this chapter, compared with the others, we humbly conceive Mr. B. has left himself but small space for the performance of the following great promises.

"I will relate *several cases* of this formidable disease; detail the appearances which presented themselves upon dissection; *investigate its nature;* point out that which I conceive to be the most judicious mode of treating it; and, finally, give such directions as will, I hope, if steadily pursued, preserve many from becoming its victims."

All this, too, in nineteen pages of modern octavo!

How happy for the shades of Whytt, Quin, Fothergill, Withering, &c. &c. that the tomb conceals them from the disgrace which here awaits them! And how must Cheyne and Yeats blush for having imposed on the world, *ech a volume,* when nineteen or twenty pages were amply sufficient!

The first case of hydrencephalus, succeeding a blow, (in a boy of 15) was mistaken (oh, shame Mr. Bedingfield!) for "*cynanche parotidea,*" and the cure was commenced by an *emetic!* Purgatives, venæsection, blisters, and mercury were afterwards employed in vain. He died on the fifteenth day, with delirium, black fœtid stools, strabismus, and dilated pupils. Post mortem examination shewed water in the lateral and third ventricles; but no structural disease in the viscera of the thorax or abdomen.

The second case of hydrencephalus succeeded amputation in a scrofulous boy of 17, and exhibited on dissection the same appearances as the first. The third succeeded an operation for the harelip in a child 3 years of age; no dissection. The fourth was successful. And as such cases are somewhat rare, we shall give it entire.

"A sickly child, 4 years of age, with deformed, rickety limbs, was placed under the care of the surgeon of the week. A few days after her admission, she was attacked with all the symptoms commonly attendant upon hydrocephalus internus. There *was* [were] also great acceleration of the pulse and a hot skin. She screamed at intervals, and refused all kinds of food. Two grains of calomel, combined with eight of jalap were given to her, and afterwards a grain of calomel every six hours. On the following morning she appeared somewhat better; her pulse was slower, and her skin more cool. The body was directed to be sponged, and the cathartic and calomel to be repeated. The next day she appeared much better; she had voided with her fæces two lumbrici, about four inches in length each; she took a little nutriment, and did not complain of her head; the pupils were less dilated; the powder and calomel were repeated. She passed on the following day, another worm, and from this time gradually recovered." Page 17.

Do the respectable physicians of the Bristol Infirmary believe, that this was a case of hydrencephalus? Do they think, that water was effused in the brain in this case? If they do, we blush for them! But we do not accuse them of such ignorance.

The fifth and last case, occurred in a boy who had psoas abscess, and was evidently a metastasis to the brain.

"The brain exhibited evident marks of active vascular action having existed. The ventricles did not contain so large a quantity of water as was expected."

From these our author builds his theory or pathology of the disease. He conceives the deposition of serous fluid to be the consequence of sympathetic inflammation of the brain; the predisposing cause being "an irritability of mind," most frequently met with in scrofulous habits; "local irritation is the immediate cause of the disease;" viz. in the first case, a blow; in the second, amputation; in the third, the operation for harelip; in the fourth, worms; in the fifth, psoas abscess: and thus ends the theory of hydrencephalus!

We were going to remark, that our author must have been acquainted with the opinions of Cheyne, Curry, Yeats, &c. who have written to shew, that the origin of hydrocephalus is generally an irritation of a remote part, as of the abdominal viscera, &c.; but we recollected, that Mr. Bedingfield's reading was confined to Cullen and the London Medical Repository. In the *latter* he must have read the review of Dr. Yeats's work, as well as Dr. Smyth's, and shews his learning accordingly in a *note*, by changing hydrocephalus into hydrencephalus.

In Mr. Bedingfield's *treatment* there is nothing but what the merest sciolist could repeat without difficulty. Tranquillity of mind; and attention to the digestive organs, are the preventives. Removal of local irritation; mercurial purgatives; bleeding; erect position; cold lotions to the head, are the remedies for the inflammatory stage:—mercury, when effusion has taken place. We ask our readers if there be a particle of information here which they did not know before. The ostrich thrusts his head into a bush, and thinks no one can see more than himself: Mr. Bedingfield publishes the practice of the Bristol Infirmary, and thinks no one ever saw such things before!

Chap. 5, page 29, on apoplexy, contains twenty-two lines, and informs us, that in sanguineous apoplexy we may bleed, but in serous, we had better let it alone. This is a "Compendium of medical practice" with a vengeance! Those who read our histories of apoplexy from Morgagni, in another department of this Journal, will entertain a curious opinion of Mr. Bedingfield's Compendium!

Chap. 6. *Hemiplegia*, p. 31 to 36. This chapter opens with a violent Philippic against the old women of the Faculty, who "still persist in administering stimulating medicines internally, and in applying rubefacients, electric sparks, &c. to the parts deprived of muscular motion." We believe, that few of our professional brethren, who know any thing of the "seat and cause" of hemiplegia would act as above. Mr. B. recommends bleeding from the arm or jugular vein, as a contrast to "the intricate, dark, and often dangerous paths of our forefathers," although, since the days of Morgagni, the measure alluded to has been employed. The excitement of nausea by emetic tartar, he thinks, should be kept up for days, and even weeks, to produce absorption. To which may be added blisters, purgatives, &c. One case is related which terminated fatally. A serous effusion was found in the ventricles and at the base of the brain.

Chap. 7. *Phrenitis*, p. 37, 38. Mr. B. witnessed several violent cases of phrenitis, all of which terminated favourably. Seldom less than twenty ounces of blood were drawn from the arm or jugular, sometimes three, four, or five times a-day. No regard was paid to quantity, but to the alleviation of symptoms. Antiphlogistic regimen; active purgatives; evaporating lotions to the shaven head, were the auxiliaries.

Chap. 8. *Inflammation of the pia mater*, p. 39, 42. A young woman, for fourteen days, had complained of violent head-ache, with dry skin, quick pulse, furred tongue, intolerance of light, and inflamed eyes. The evening after admission delirium came on. Third day she muttered incessantly. Fourth day she lay on her side, and kept moving in a manner difficult to describe Fifth day, comatose, fæces and urine passing involuntarily. "*See-saw motion of the body continued.*" Ninth day, life terminated by an apoplectic paroxysm.—*Sectio cadaveris.* Sinuses of the dura mater full of blood. Half an ounce of serum in the lateral ventricles. At the basis of the cranium a large quantity of serum tinged with blood. The arachnoid coat thickened, not transparent. A jelly-like substance near the decussation of the optic nerves. "The tuber annulare, crura cerebri et cerebelli were almost veiled from the eye," by inflammation and thickening of the pia mater.

"Was the peculiar motion," says Mr. B. "to which I alluded, occasioned by the tuber annulare and its continuations participating in the inflammatory process with which the pia mater invest-ing them was attacked?"

To this *ingenious* question, we are sorry that we cannot return a *sagacious* answer; since we are not so far initiated into the mysteries of the Gall and Spurzheim theory, as to determine with precision, whether the "*see-saw*" *faculty* be situated in the tuber annulare.

The 8th Chapter contains the history of rather an *Hibernian* kind of *abscess* in the brain, where "the walls of the cavity seemed to be without organization," and where "there was *no pus or other fluid!*" What became of the pus of this abscess; or what reason Mr. B. had for believing there ever was pus there, is no where stated! The disorganization, however, went to a great extent before the patient's life was terminated.

The 9th Chap. on Epilepsy, 47, 50, offers little consoling in this obstinate disease. To diminish the force of the arterial system by drawing small quantities of blood from the arm; to give an active purgative once or twice a week, and on the intermediate days, the oxide of zinc in doses to excite nausea, appeared the most successful plan at the Bristol Dispensary.

Chap. 10. *Chorea*, p. 51, 53. Thirty-nine cases of this disease, out of forty that presented themselves, were cured by the oxide of zinc, given in five grain doses three times a-day, gradually increasing the quantity to a scruple.

"So speedily and decidedly did this remedy put a stop to the disease, that I cannot avoid regarding it as a specific for it."

We wish Mr. Bedingfield's assertions may prove true.

Chap. 11. *Tetanus*, p. 53, 57. Only one case was witnessed by Mr. Bedingfield, and this proved fatal. The detail of it, however, was more fortunate, for it formed a whole chapter for the "Compendium of Medical Practice."

Chap. 12. *Tic Douloureux.* This chapter contains a novel fact, and probably an useful hint, viz. that "of paralysing the nerves by the application of cerussa," first suggested by a pupil in London. It succeeded in a case under Astley Cooper that had resisted every other remedy, and even the knife.

"Two scruples formed into an ointment, were rubbed in the morning on the affected cheek, about an hour before the paroxysm of the disease was expected. This application was continued for a month or more, and the man left the hospital apparently perfectly cured."

The effect of the lead is represented as rapid and striking, the patient being rendered comparatively comfortable in a short time, from a state of excruciating torment. It produced no particular effect on the bowels or system.

Chap. 13. *The functions of the brain*, p. 61, 72. Had our author exercised the functions of his brain in *reading* before he began to *write*, he would have saved himself much embarrassment. A man, to excite public attention to his writings, in these days, without reading or study on his own part, must be a genius of very different mould from Mr. Bedingfield. Who would expect, that the author of a medical work in the year 1816, would make the following confession.

"At the time of writing it, I had neither heard nor *read*, that the brain had been regarded as a *glandular* organ." p. 61.

Had none of the physicians of the Bristol Infirmary the charity to lend Mr. Bedingfield Parr's Medical Dictionary, while they—"encouraged his attempts and cheered his labours," in the present performance? When searching Cullen's *Nosology* for the definitions at the top of his chapters, could he not have extended his researches to that author's "*first lines*," where he would have *read* of the brain as a *glandular* organ? But Parr is the author we recommend to Mr. B. He will there be astonished to find every thing which is known at the Bristol Infirmary, and perhaps more.—Mr. Bedingfield's theory is:—"that the office of the brain is to convert a portion of the blood sent to it, into a fluid, in which the living principle essentially resides; that this fluid is distributed by the nerves to every part of the body, &c." p. 61. Thus have we the *old* doctrine of "nervous fluid" "animal spirits," &c. secreted in the brain and distributed through the nerves, brought forward in the 19th Century as a *new* discovery!

"It was for a *long time* supposed, that the brain was a *gland* which secreted a fine fluid, conveyed through the minute fibres of the nerves, &c." *Parr. Art. Cerebrum.*

The most beautiful and original *illustration* of this theory, is contained in the following passage:

"The soul, with *its* appendage, the nervous system, may be aptly compared to *a spider upon his web.* 'Touch the web ever so lightly, and the animal is conscious of it; make an impression upon a nerve, a quantity of fluid *passes off;* by the escape of the fluid the soul is informed, that an impression has been made.' 70.

But enough of the Bristol theory.

SECT. II. CHAP. 1. *Ophthalmia,* p. 72, 73. The best practice, Mr. B. says, is effected by frequent scarifications of the conjunctiva; watery solutions of opium; and where there is symptomatic fever, &c. general depletion; by bleeding, purging, and the antiphlogistic regimen. Light is of course to be excluded.

CHAP. 2. *Dentitio,* 74, 76. We believe with Mr. B. that dentitio difficilis often "lays the fonndation for diseases in their nature the most formidable."

"Upon a free division of the gums we must chiefly rely." "In less than two hours after the operation, I have frequently seen the most violent symptoms diminished; hydrocephalus arrested in its progress; convulsions cease; symptomatic fever subside: and, in a few days, cutaneous eruptions disappear."

CHAP. 3. *Odontalgia,* 77, 78. We shall extract the following Recipe for the Tooth-ache.

"Put one or two drachms of henbane-seed upon a red hot iron, or some lighted cinders, and immediately cover them over with a bason. As soon as you suppose the seed to be consumed, and the vessel impregnated with the fumes; place it upon its bottom and fill it with boiling water. The person affected with the tooth-ach is then to inhale the vapour for twenty minutes or half an hour, a blanket or other covering being thrown over the head and shoulders to prevent its escape."

The next short Chapter on Cynanche, contains nothing novel, except a remark which we know to be often true; namely, that gargles seldom have much good effect in these inflammations; and that, "the inhalation of the steam of hot vinegar, or the gradual deglutition of small quantities of honey acidulated with the muriatic acid, will be found more effectual in allaying irritation, or in giving tone to whatever parts are in a state of relaxation."

CHAP. 5. *Pharyngis ulceratio.* This Chapter contains the particulars of rather an interesting case of guttural disease. A woman, 30 years of age, was apparently cured of pneumonia by the usual means, when she began to complain of uneasy sensations in the throat, attended with a disposition to cough, and a frequent discharge of frothy mucus. To these were soon added, hoarseness; loss of voice; difficulty of deglutition and respiration; the former with much pain. Pain was also felt on handling the thyroid cartilage. No disease could be discovered by inspection of the fauces. In twelve weeks she sank exhausted.

*Sectio cadaveris.* Membrane of the larynx thickened; diameter of the rima glottidis much diminished; trachea *naturally* very small; just below the arytenoid cartilages, a considerable degree of inflammation, extending an inch and a half along the posterior part of the trachea. At the anterior part of the pharynx, just below the rima glottidis, a tumor, in shape and size resembling a filbert was situated. The portion of it in contact with the pharynx, and contiguous to the larynx, ulcerated.

CHAP. 6. *Laryngis ulceratio,* p. 86, 88. Although we believe with Mr. B. that this disease is of more frequent occurrence than is generally suspected; yet we cannot agree that, "it is *often* mistaken for phthisis pulmonalis," except by those *bunglers* in medicine whom *experience* has rivetted in error, and who are "dead in trespasses and sins" against every law of diagnosis!

*Case* 1. W. B. aged 39, was several times admitted for pneumonia in the course of three months, and cured. A tickling sensation about the throat; cough; and ultimately hoarseness remained. To these were added afterwards, incessant, harrassing cough; copious expectoration of frothy mucus, interspersed with purulent particles; pulse frequent and rather full; tongue white in the centre, florid at the edges; uvula elongated; back part of fauces very red; tonsils somewhat enlarged; appetite and spirits good; lassitude, and want of rest at night; emaciation; skin dry and sometimes hot; cheeks sometimes flushed; never perspired properly. In four months he sunk exhausted.

*Sectio cadaveris.* Superior aperture of the larynx diminished; its membrane thickened and vascular. One of the arytenoid cartilages completely, and the other partially destroyed by an ulcer. No marks of the disease in the trachea.

*Case* 2. Sarah Hopkins, aged 19, admitted the 2d of June 1814, affected with difficult respiration, accompanied by a croupy noise; cough; soreness of the throat; elongation of the uvula; enlargement of the tonsils. No constitutional disease; pulse natural; chest free from pain. Illness of three months standing.

*Treatment.* Venæsection; blister to the chest; saline antimonial medicines; linctus for the cough. She died suddenly three days after admission.

*Sectio cadaveris.* The rima glottidis very much contracted; the epiglottis abraded. Internal surface of the larynx and trachea ulcerated; the latter filled with purulent matter. Lungs, heart, and abdominal viscera sound.

Every case of ulcerated *pharynx*, larynx, and trachea, that has fallen under Mr. B's observation, has terminated fatally. Surely Mr. B. must have seen few *pharyngeal* ulcerations; for they are by no means so generally mortal, as here described. That the other species are extremely dangerous we are well aware; but we think Mr. B's proposal "to make an opening into the trachea or larynx, and apply such substances to the ulcer as would have the effect of exciting a healthy action upon its surface," a most rash and hazardous operation, and extremely unlikely to do any good; but rather to accelerate the fatal catastrophe.

Chap. 8. *Bronchitis.* G. C. aged 40 years, after exposure to great heat in a sugar-house, went into the open air, and was soon after attacked with symptoms of pneumonia. Fourteen days after the attack, he was admitted into the Infirmary with the following symptoms:—Great pain in the chest, and about the scrobiculis cordis; dyspnœa; inability to lie on either sides or back; could only remain in an upright posture, resting his head on his hands and knees, the trunk bent forward; violent cough; purulo-mucous expectoration; scanty urine; pulse extremely rapid; countenance indicative of thoracic effusion. Bleeding, blistering, purgatives, diuretics, mercury, produced no relief. He died four days after admission.

*Sectio cadaveris.* Lungs externally, heart, pleura, pericardium, sound: no adhesions, no effusion. Abdominal viscera healthy. Internal membrane of the trachea, bronchia, and their ramifications inflamed in a high degree. Bronchia filled with purulent secretion from these surfaces. No vestige of abscess in the substance of the lungs.

Chap. 9. *Hæmoptysis*, p. 99, 105. This is rather an interesting chapter. Mr. B. justly considers venæsection as our sheet anchor in hæmoptysis. After bleeding, digitalis is sometimes useful; but Mr. B. anathematises the use of this in the most determined manner, of which, more presently. After rigid abstinence, cool air, cold drinks, &c. have been enjoined, Mr. B. recommends a small quantity of syrup of opium or white poppy, to be held in the mouth, and slowly swallowed, to allay the irritation at the upper part of the trachea and the disposition to cough. Infus. rosæ and sulph. acid of course, and half a grain or a grain of super-acetate of lead, taken twice or thrice a day. We have found this best combined with opium, in all cases where it was deemed advisable to employ it. Blister to the chest. If these means fail, full vomiting, and subsequent nausea are to be excited by ipecacuanha or emetic tartar. Mr. B. has sometimes seen benefit from holding a piece of alum in the mouth for a considerable time.

In respect to digitalis, Mr. B. is of opinion that the good derived from it bears no proportion to the mischief it produces.

"Unless given in sufficient doses to produce its specific effects, it frequently increases the force of the circulation, and rapidity of the pulse. If administered in quantities large enough to bring the system under its influence, it will, sometimes, in spite of all our vigilance, destroy life." 100.

The first part of this passage is not original, as Mr. B. would have known had he been in the habit of reading as well as writing. Dr. Saunders, of Edinburgh, several years ago, published a volume, to prove the stimulating effects of digitalis on the vascular system. As to the deleterious effects so much deprecated here, we cannot confirm them by our experience, though we have used the medicine and seen it used on a pretty extensive scale. As some caution, however, is necessary in its administration, we shall give one of Mr. B's. cases, wherein it is accused of destroying life.

"A woman, 22 years of age, labouring under symptoms of acute pneumonia, and a supposed effusion into the cavity of the thorax, after a moderate bleeding, was directed to take half an ounce of infus. digital. every six hours. She took three doses, and died in the act of raising herself from the pillow. Upon dissection, traces of inflammation were found within the lungs, and upon the pleura, but they were not at all sufficient to account for the death of the patient. No effusion into the thoracic cavity had taken place. The inner coat of the stomach was free from any peculiar diseased appearance. The abdominal viscera sound." 100.

Although it appears *probable* that the digitalis caused the death of this patient; yet, as the head was not examined, it is by no means *certain* that the fatal event was attributable to the medicine.

The following are Mr. B's objections to digitalis:

"Upon some persons, it acts as a powerful and deadly poison; but, upon what peculiarities of constitution this depends, we are at present ignorant.

"If not given in sufficient quantity to produce its specific effects, it frequently increases the inflammatory diathesis. When the habit is once brought under its influence, it will continue for an indefinite period.

"Should the effect produced be too powerful, we are without ability to moderate it. The life of a man, under the influence of this medicine, is in perpetual jeopardy; the incautious, or accidental elevation of his body may destroy him.

"It may sometimes be given to a great extent before it produces any effect; its presence will then be suddenly manifested, and perhaps so powerfully as to extinguish the vital spark.

"Lastly, I object to digitalis, because I firmly believe, that all its beneficial effects may be derived from less powerful and less dangerous ingredients. Among these I number ipecacuan, squills, and tartarized antimony, given in nauseating doses."

We are of opinion, that Mr. B. has here much exaggerated the danger, the uncertainty, and the unmanageable nature of this medicine. Were it such as he has represented it to be, the medical world would have, ere this, proscribed it from the Materia Medica. Nevertheless, we should act with caution and *vigilance* in its administration.*

Mr. B's short Chapter on Phthisis Pulmon. offers nothing but his belief that the disease is incurable.

Chap. 11. *Pneumonia.* Mr. B. is of opinion that bleeding, *pleno rivo,* in this disease is often injurious, as

---

* "He who does not attentively watch the state of the pulse, the stomach, the bowels, and the sensorial functions, should never prescribe it. But if these be carefully watched, and the medicine withdrawn as soon as any of them are materially affected, I hesitate not to affirm, that no serious inconvenience will ever ensue from it, and that it may be administered with as much safety as any of the more active medicines in daily use."—*M'Lean on Hydrothorax,* p. 172.—The following was the form which Dr. M'Lean generally commenced with:

℞. Infusi digitalis ℥j. Aq. menth. pip. ʒiij. Carb. potass, gr. x, ad ℈j spt. æth. nit. ʒß, ad ʒj. ft. haustus bis terve in die sumendus.

The Reader will balance these two authorities, and recollect that the general voice of the Profession is in favour of Dr. M'Lean's statement.

it induces syncope before the vascular system is sufficiently depleted. To this, we would answer, that the very circumstance of syncope itself is highly favourable, as it checks most powerfully the action of inflammation in any viscus. As for the patient being left "sixteen or twenty-four hours" till the Physician returns the next day, while inflammation exists in so important an organ as that of respiration, it is preposterous. He should be seen every three or four hours, and the vein opened as the pulse rose or the pain increased. Treated in this manner, we should have no hesitation in adhering to the plan of bleeding from a large orifice.

Chap. 12. *Empyema.* A long case is here related of a man, in whom an imposthume took place, and where the operation was performed, but without success.

Chap. 13. *Hydrothorax.* Mr. Bedingfield is a strenuous advocate for the operation of paracentesis thoracis, in this disease, when squills, digitalis, elaterium, mercury, &c. fail. We have so often found ourselves deceived in the *site* of the water in hydrothorax, that we should be extremely averse to the operation. It is next to impossible, to ascertain whether the water is *solely* in the thoracic cavities, or in the pericardium. It is true, that Mr. B. makes no difficulty in finding out the time and place for the operation.

"The symptoms of the disease, together with an attentive examination of the chest, in the manner directed by Corvisart, will, *for the most part*, enable us to determine whether or not water be present, *as well as the point* at which the opening may be most advantageously made." 125.

Lest Mr. B. might think us harsh, we shall reply to this in the words of Vieussens.

"It is *not* so easy to know the dropsy of the thorax as some physicians believe, who *ascribe too much to themselves;* for those who have inspected *many bodies*, have at least learnt to *doubt*, when the others, who do not take this trouble, *are in no doubt at all.*"—Traite' du Cœur.

Our own countryman, Dr. M'Lean, whose opinion on this subject is entitled to some weight, observes, that "it is here to be lamented, that the various morbid alterations of structure of the heart itself, of its valves, large vessels, or other organs within the cavity of the chest, with which these two diseases (hydrothorax and hydrocardia) are *generally connected,* and by which they are *often produced,* render the diagnosis *much more difficult,* since the symptoms peculiar to each of these, will be obscured by being blended together."* And again, at page 70, " it must be confessed, that no one has as yet been fortunate in pointing out *any individual sign* which may be said to belong exclusively to the one or the other."

As for hydrothorax and empyema, they bear no analogy to each other whatever; yet it is because that in the *latter* the operation is sometimes successful, Mr. B. builds his arguments in favour of the same operation in hydrothorax!

" Squills, says our author, in nauseating doses, will be found serviceable; but elaterium, administered in sufficient quantities to excite frequent, copious, and watery evacuations from the intestines, will be found more beneficial. In the early stages of the disease, venæsection will be, not merely proper, but indispensable." 127.

Favourable as we are to the *depleting* system in most diseases, we cannot but censure this indiscriminate employment of the lancet in hydrothorax. That there are states of the vascular system, wherein the accumulation of blood in the right side of the heart and in the head, threatening suffocation, requires venæsection, we know; and the necessity for such a measure will be evinced by a livid, swollen aspect; dyspnœa and distension of the jugular and other veins; full, hard pulse, and palpitation of the heart. Here a small venæsection (and a very small one will suffice) may save the life of the patient. But, surely, the sweeping position of Mr. Bedingfield respecting venæsection is highly indicative of inexperience in this disease. The venerable Hippocrates employed blood-letting in dropsy, but with caution and restrictions.

" Si hydropicus *difficulter* spiraverit, et tempus vernum fuerit, et simul *ætas viguerit* et virium robor adsit, de brachio sanguinem oportet." *De Dieta.*

SECT. III. CHAP. 1. *Carditis.* In almost all the cases of cardiac inflammation which Mr. B. has seen, it has either been combined, or has alternated with, acute rheumatism. The following is the case which constitutes this chapter:—A baker, aged 18 years, felt a violent fluttering at his heart on going to bed. It appeared to beat

---

* On hydrothorax, p. 66.—This work, so unaccountably neglected, we would recommend to the library of every medical gentleman. We would particularly recommend its perusal to Mr. B. who will probably alter his opinion respecting paracent. thorac. in consequence.

twenty times quicker than usual. This was attended with violent pain in the chest, spitting of blood, sickness at stomach. On the four following days his limbs became stiff, cold, and painful; legs swelled, fingers contracted.—5th day. Admitted into the Infirmary with the following symptoms:—Great fluttering of the heart, attended with a rapid, irregular, and scarcely distinguishable pulse. Breathing extremely laborious; expectoration of mucus tinged with blood; costive bowels.

*Treatment.* Blister to the chest; two grains of digitalis every night; saline antimonial mixture, with syrup pap. alb. in the day.—6th day. The digitalis excited great nausea, and was omitted; the mixture continued.—7th day. The increased action of the heart continued; pulse full and irregular; bowels constipated. Another blister applied to the chest; ten ounces of blood from the arm; a purgative enema.—7th day. The blood rather buffy: the enema had operated; venæsectio ad ℥xvi.—8th day. Blood not buffed; pulse hard, quick, irregular, 160 in the minute. A grain and a half of pulv. digit. every four hours, with the saline mixture.—9th day. Symptoms as yesterday. Digitalis to be continued; eight grains of aloes, and one and a half of opium, at bed-time.—10th day. Great amelioration of all the violent symptoms. Pulse 80, regular and full. Complains of pain in the shoulder blades, which he now says he felt throughout the attack.—11th day. No alteration since yesterday. Continue the same medicine.

From this time he regularly improved, and left the hospital in a fortnight afterwards, in a state of convalescence.

Mr. B. thinks, and we agree with him perfectly, that venæsection was not carried here to a proper extent at the beginning of the disease. He believes that our principal remedies in carditis are copious venæsection and opium.

A case of carditis fell under Mr. Bedingfield's observation, wherein the whole arterial system seemed to participate. A violent and rapid pulsation could be seen as well as felt, in its minute ramifications. It was peculiarly distinguishable in the labial and digital arteries. This man lost, in twelve days, 260 ounces of blood. For eleven days in succession, twenty ounces were abstracted; and, on the 12th day, 40 ounces at one operation.

"From this last bleeding, he derived more advantage than from all those which preceded it. Syncope was induced by it, and a permanent diminution in the force of the heart and arteries was effected."

He recovered perfectly. A violent and most profuse perspiration was a conspicuous symptom in this man's case. 133.

( To be continued in our next Number. )

